# New paradigm in the economic literature on global warming

**DOI:** 10.1016/j.heliyon.2023.e17715

**Published:** 2023-07-05

**Authors:** Miguel Cuerdo-Mir, Raquel Ibar-Alonso

**Affiliations:** Department of Applied Economics I and History and Economic Institutions, Universidad Rey Juan Carlos, P° Artilleros s/n, 28032, Madrid, Spain

**Keywords:** Economics of climate change, Climate finance, Climate change corporate, Climate capitalism, Climate gateway belief, Scientometric analysis

## Abstract

This study examines how international agreements (especially the 1992 Rio Summit, the 1997 Kyoto Protocol, and the 2015 Paris Agreement) on climate change have fueled rampant economic literature worldwide. However, it has not been systematically classified or distinguished from the more traditional studies in this field. Hence, we use a scientometric analysis using four different approaches: natural language processing (NLP), citation analysis, co-citation network analysis, and content analysis. We conduct an ambitious Boolean search of 30 terms in Scopus and use NLP, along with unsupervised statistical learning techniques and content analysis to classify and analyze 2400 of the most relevant studies in this field. As such, independent results are complementary. We provide novel literature by mapping four major clusters: climate change corporate, climate finance, climate capitalism, and climate gateway belief.

## Highlights

• Four emerging research fields with their own identity are identified: climate capitalism, sustainable corporate, gateway belief, and climate finance.
• There is a specialized field of sustainable development, in which the multisystemic vision has been lost.
• This classification yields a differentiated profile for the business literature on sustainability, which has modified the more traditional conception of corporate social responsibility.
• Green finance, a young field of research linked to the development of finance and the fight against climate change.

## Graphical abstract

**Figure:**
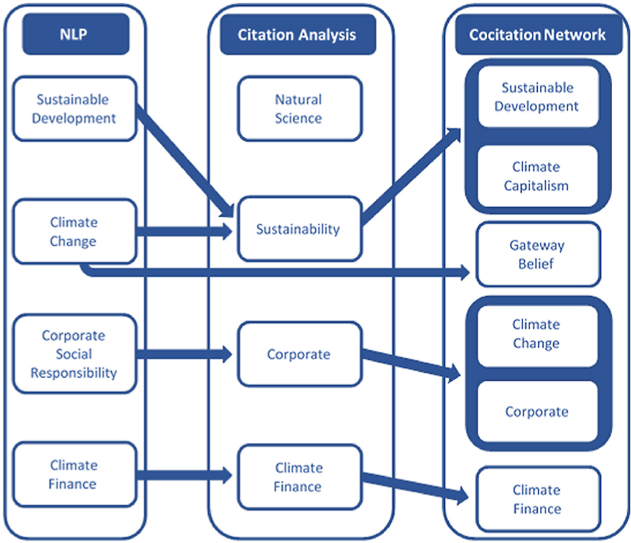

## 1 Introduction

In recent years, many scientific articles have analyzed the fight against climate change, its regulation, its financing, and its consequences from an economic perspective. However, to date, this surge in scientific production has not been dealt with in a systematic way that would allow us to know which new scientific fields or methodological orientations are being followed in most cases.

The goal of the present research is to identify and classify the different fields of research that compose the recent economic literature on the global issue of climate change. For this purpose, it uses advances in natural language processing (NLP) because they have allowed the traditional systematization, ordering, and analysis of relevant aspects of the scientific literature to undergo significant changes in recent years. The study's main contribution is considering this approach as a novel starting point in determining the characteristics of classified elements and identifying whether they are sufficiently important to be proposed as new or different fields of research within the economic literature.

We evaluated the most relevant and recent studies using this approach for the economic analysis of sustainability and climate change and found that the use of these methodologies has significantly contributed over the past decade. Several of the most important studies of the last years focused on different topics related to sustainability or climate change economics, as shown in Table 1, attempt to systematize the economic literature related to a particular aspect of sustainability or climate change. All of them, and many others, used a common bibliometric approach based on NLP techniques. Some of them combined these methods with network theory or statistical or econometric analyses of the measurement and characteristics of the citations and bibliographic references.

**Table 1:** Last Published Papers using scientometric analysis, networks theory, or literature analysis through database focused on the economics of sustainability and climate change.

Works	Techniques used	Field of Interest
Olawumi, & Chan, 2018	Scientometric review	Global Research of Sustainability
Zhang, Zhang, & Managi, 2019	Bibliometric Analysis	Green Finance
Tang, Zhang, Liu, & Wu, 2020	Scientometric review	Systemic Risk and Macroprudential Policy
Ballestar, Cuerdo-Mir, & Freire-Rubio, 2020	Scientometrics and Networks Theory	Sustainability in Science and Social Networks
Li, & Li, 2021	Scientometric Analysis and Literature Review	Risk Governance and Sustainability
Wu, Jiang, Li, & Zhang, 2021	Scientometric Study	Urban Sustainability

Table 1 Last Published Papers using scientometric analysis, networks theory, or literature analysis through database focused on the economics of sustainability and climate change [[1], [2], [3], [4], [5], [6]].

Among them and the closest to our proposal, Olawumi and Chan [1]), using scientometric techniques such as co-author analysis, co-word analysis, clusters, or geospatial analysis, study “the status quo and trend in the sustainability research field”, and it is an important step forward in the understanding of a holistic and comprehensive concept of sustainability. Probably because of the choice of terms, periods, and focus, none of the clusters identified refers specifically to the economics literature on climate change as an emerging discipline. The case of Zhang, Zhang, and Managi's [3] paper is focused only on sustainable finance literature but is very relevant because it leaves no doubt that there is an emerging literature on sustainable finance. Ballester, Cuerdo-Mir, and Freire-Rubio [6] are the first analyzing the concept of “sustainability” using data collected from social media and apply an innovative collection of technologies and analytic methods. It reflects that social listening is a powerful method to analyze social phenomena of relevance as climate change threats, but does not show a clear interest in exploring the different approaches from which academics can approach the issue. In other cases, as Wu, Jiang, Li, and Zhang's [4] paper, the topic, using the scientometric package and output visualization results for analysis and discussion, is focused on the literature on how to manage climate change problems in smart cities. Even though the specific interest of the study, the clusters resulting from the study highlight the importance of these methodologies of analysis to systematize fields of study of interest and well-defined when we face problems of sustainable development such as those related to climate change. Li and Li's [5,6] paper analyzes through scientometric methods the relationships between risk governance and sustainability, underlying “the heterogeneity of the risk governance and sustainability research” and introducing the important question of a literature that needs to be interdisciplinary when studying risk governance environment-oriented.

However, despite the importance of the subject, none of them faces aa systematic analysis of the growing body of whole economic literature on climate change. Therefore, having studied these contributions and starting from their own limitations, we propose a perspective that entails a thorough and explicit review of this literature stream and its purposes. In our approach, using NLP techniques and the tools outlined above and explained in detail below, this scientometric approach seeks to answer an open set of questions related to the socio-economic analysis of climate change. Among these, the presence of a current economic literature stream on climate change is the most obvious. We also explore concerns such as when and why this literature came into being, where it came from, how it is developing, what issues it addresses, what type of problems it is trying to solve, who the key authors are, and which are the seminal articles.

The research questions framed in this study are as follows:

1. Is it possible to sort through the vast literature on the economics of climate change?
2. Is the new literature stream on the economics of climate change reductionist relative to the pre-Paris Agreement environmental economics literature?
3. Has the low-carbon economy goal favored the emergence of a distinct business economics literature, changing the concept of corporate social responsibility?
4. Have the agreements on combating climate change from 2015 onward led to differentiated green finance literature?

## 2 Methodology

This study follows a mixed methodological approach combining bibliometric and scientometric analysis and a comprehensive qualitative review for a deep understanding of the climate change literature. Torraco [7] identifies five different purposes to conduct a bibliometric review: to carry out a historical review of the literature, analyze the literature using statistical tools such as clustering and network analysis, synthesize the existing literature, in-depth study of a subject and re-conceptualize it through new findings, and seek answers for research hypotheses proposed on a specific subject. In this study, these five objectives were considered to respond to the research hypotheses, providing robustness to the conclusions.

Additionally, the methodology used contemplates three possible approaches to literature review: bibliometric analysis, systematic literature review, and narrative review [[8], [9], [10]]. Initially, we conducted an exhaustive and systematic search of publications on the economics of climate change. Throughout the research process, a detailed content analysis of the most relevant publications (narrative review) was conducted, leading to a critical evaluation and synthesis of the sub-areas of knowledge (systematic review), framed within the overall theme. Mathematical and statistical algorithms were applied to study the behavior and evolution of publications, and the relationships between authors and the studies was extracted from Scopus (bibliometric analysis).

The methodology proposed in this study combines the use of quantitative and qualitative statistical tools on the same database. Therefore, we could analyze the specialized literature based on scientometrics and carry out a content analysis of the most relevant documents from different perspectives of scientific interest.

Consequently, the methodological phases of this research are as follows. First, the documents indexed in the Scopus database were selected based on Boolean advanced search techniques [11] in the field of climate change (see Section 3.1).

Second, NLP was used to conduct a bibliometric analysis of the extracted documents. The database was created and refined with the R-Studio software version 1.2.5033 and analyses were conducted using the Bibliometrix tool and algorithm programming in R language version 4.1.0 [12]. To accurately analyze the abstracts, some actions were carried out to clean up the information collected in the database. Especially, to tokenize the text from n-grams (n = 1, 2), and eliminate words that lack meaning by themselves with the use of the “stopwords” dictionary of the “NLP” library contained in the NLP package version 0.2-1.

Third, we conducted an analysis of the frequency distribution of the identified n-grams in the keywords. The repetition of terms was counted and represented by word clouds, and the evolution of the most frequent concepts was studied throughout the analysis period. The terms used in the search were not included in the list of the most frequent keywords, as they were removed from the graph to avoid obscuring the list of the most repeated new terms.

Fourth, we studied the thematic evolution of the literature based on the n-grams extracted from the abstracts. For this purpose, we carried out a dynamic cluster analysis [13], in which the groups identified the underlying thematic areas in the field of research. Dynamic clustering combines the accuracy with which the clustering result captures the network structure, and the similarity between the current clustering result and the previous cluster result. This temporal smoothing allows to control the balance between temporal noise and the true conceptual drift of the temporal patterns. The centrality and density of the clusters found were calculated [14]. Centrality analyzes the external links between the different thematic areas, measuring the importance of a topic within the overall context of the research. Density measures the intensity of the internal linkages in each identified thematic area and provides information on the overall development of each cluster. Following Callon, Courtial and Laville [15], the joint use of certain terms allows us to see their position as a combination of centrality - to what extent these terms are able to synthesize the problems considered crucial in the research - and density - how strong the links between words that make up the corresponding cluster are and, to see the coherence of the cluster and its capacity for development.

Fifth, to complement previous analyses, the content analysis of the most relevant research terms, extracted in the first phase for the most cited documents, was carried out. For this purpose, we conducted an exhaustive reading and text analysis of the most relevant articles in each of the latent thematic sub-areas of climate change. The information extracted with this qualitative technique is crucial in understanding the relationships in the statistical analyses.

Finally, a co-citation network of the references of the publications in the database was built [16]. Although different algorithms (clustering methods) exist to identify these subcomponents, in this study a graphical model of all networks was created using Louvain community detection algorithm [17] implemented in the networkPlot function of the R package. This method is well suited to weighted graphs, as with many clustering algorithms, the quality of the partitioning is optimized by maximizing the modularity function [18]. Graph theory, applied to social networks, was used to carry out a network analysis based on the following main statistical measures: degree, betweenness, closeness, and PageRank [19]. PageRank algorithm has been adapted to bibliometric analysis to quantify the relative importance weights of a citation network [20].

This methodology makes it possible to identify the subfields of research, latent in the literature on climate change. It also detects the topics that are most relevant to understanding the birth of scientific interest in climate change, the changes they have undergone over time, and emerging topics that will become the main areas of scientific interest in the short-medium term.

## 3 Data, analysis, and results

### 3.1 Data

The search strategy followed in this study focuses on the articles published in the Scopus database because it is the largest and contains the most recent and updated publications with great relevance in the scientific field. The starting point of this research is the compilation of articles specialized in climate change based on a Boolean search. The terms used for the search were found by conducting a qualitative review of the specific literature on climate change.

**Fig. 1:**
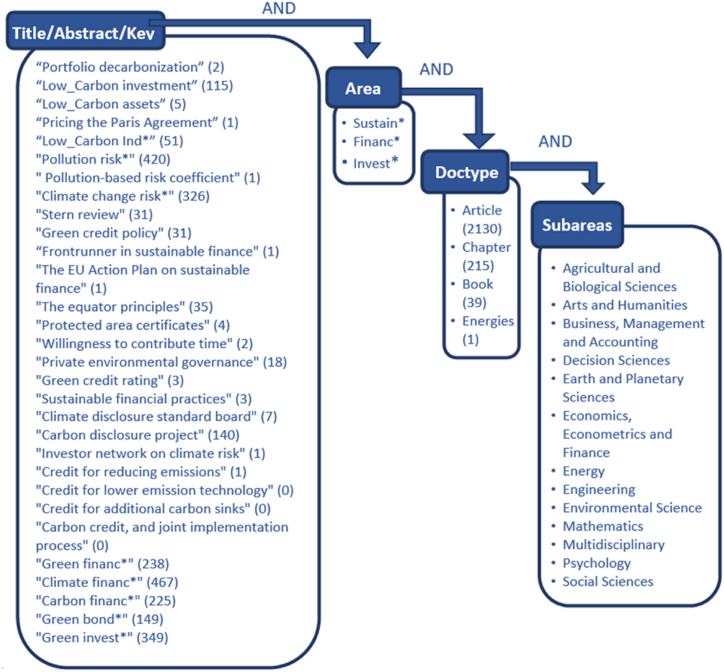
Search criteria in Scopus. The number of documents per individual search term is shown between parentheses.

By combining a list of 30 compound terms (two or more words) associated with a broad semantic field, we can circumscribe the area of study related to climate change and find the most relevant keywords from an economic viewpoint. This Boolean exercise established that any of the terms chosen must be related to a root term, such as “sustain,” “finance,” or “invest.” Additionally, to limit the search to several sub-areas of analysis, the study reduced its scope to the fields of economics, business, and finance. Finally, one extra Boolean condition was introduced to search either the title of the research, the abstract, or the keywords (Fig. 1). Therefore, we used the following the advanced search equation: TITLE-ABS-KEY((“PORTFOLIO DECARBONIZATION” OR “LOW_CARBON INVESTMENT” OR “LOW_CARBON ASSETS” OR “PRICING THE PARIS AGREEMENT” OR “LOW_CARBON IND*" OR “POLLUTION RISK*" OR “POLLUTION-BASED RISK COEFFICIENT” OR “CLIMATE CHANGE RISK*" OR “STERN REVIEW” OR “GREEN CREDIT POLICY” OR “FRONTRUNNER IN SUSTAINABLE FINANCE” OR “THE EU ACTION PLAN ON SUSTAINABLE FINANCE” OR “THE EQUATOR PRINCIPLES” OR “PROTECTED AREA CERTIFICATES” OR ″ WILLINGNESS TO CONTRIBUTE TIME” OR “PRIVATE ENVIRONMENTAL GOVERNANCE” OR “GREEN CREDIT RATING” OR “SUSTAINABLE FINANCIAL PRACTICES” OR “CLIMATE DISCLOSURE STANDARD BOARD” OR ″ CARBON DISCLOSURE PROJECT” OR “INVESTOR NETWORK ON CLIMATE RISK” OR “CREDIT FOR REDUCING EMISSIONS” OR “CREDIT FOR LOWER_EMISSION TECHNOLOGY” OR “CREDIT FOR ADDITIONAL CARBON SINKS” OR ″ CARBON CREDIT AND JOINT IMPLEMENTATION PROCESS” OR “GREEN FINANC*" or “CLIMATE FINANC*" or “CARBON FINANC*" or “GREEN BOND*" or “GREEN INVEST*”) AND (“SUSTAIN*" OR “FINANC*" OR “INVEST*")) AND (LIMIT-TO (SUBJAREA,"ENVI”) OR LIMIT-TO (SUBJAREA,"SOCI”) OR LIMIT-TO (SUBJAREA,"BUSI”) OR LIMIT-TO (SUBJAREA,"EART”) OR LIMIT-TO (SUBJAREA,"ENER”) OR LIMIT-TO (SUBJAREA,"ECON”) OR LIMIT-TO (SUBJAREA,"ENGI”) OR LIMIT-TO (SUBJAREA,"AGRI”) OR LIMIT-TO (SUBJAREA,"MATH”) OR LIMIT-TO (SUBJAREA,"DECI”) OR LIMIT-TO (SUBJAREA,"ARTS”) OR LIMIT-TO (SUBJAREA,"MULT”) OR LIMIT-TO (SUBJAREA,"PSYC”)) AND (LIMIT-TO (DOCTYPE,"ar”) OR LIMIT-TO (DOCTYPE,"ch”) OR LIMIT-TO (DOCTYPE,"bk”)).

The search was conducted on April 10, 2021, on the Scopus database (https://www.scopus.com). This combined search produced a sample of 2444 documents; 59 of which did not include abstracts or their authors were relatively unidentified. Therefore, only 2385 documents were used in the analysis [21].

**Fig. 2:**
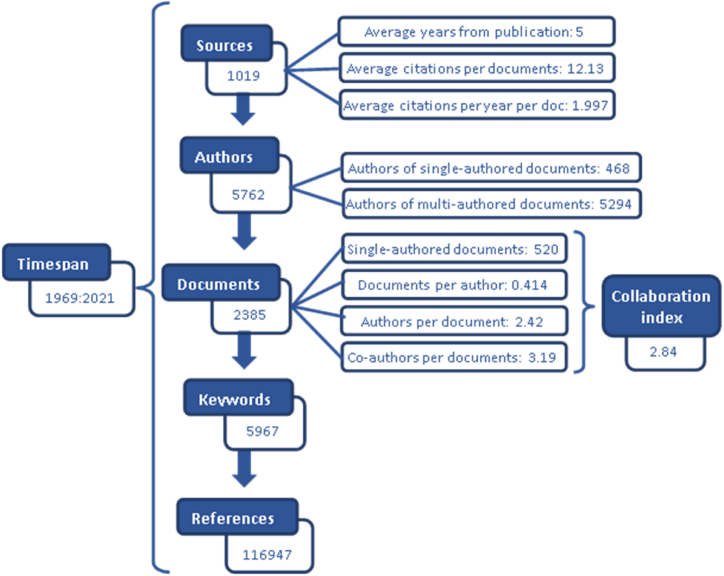
Search results.

The first result (Fig. 2) is the location of some of these terms in 1019 different publication sources, covering 2130 journals, 39 books, 215 book chapters, and 1 paper, published from 1969 to March 2021. At least one of the terms appeared across the 2385 documents, most with multiple authorship (collaboration index of 2.84), being dominated by authors who have published no more than two papers in this broad area of research. Individual authors were limited to 520 papers. Although the sample provided almost 117,000 bibliographical references, it was a very young literature stream, with an average age of 5 years and an average number of citations of just over 12.

**Fig. 3:**
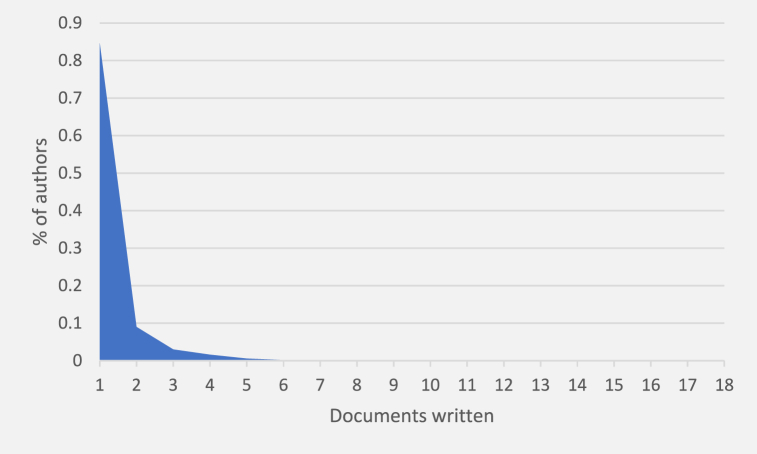
Author productivity.

An initial approximation of the productivity of the authors of these 2385 documents, illustrates (Fig. 3) that almost 85% contributed to a single document, while just over 13% collaborated in two to four documents. The number of authors participating in five or more papers was marginal. The types of literature searched mainly corresponded to authors who were not involved in the topics directly related to the search terms.

### 3.2 General evolution

**Fig. 4:**
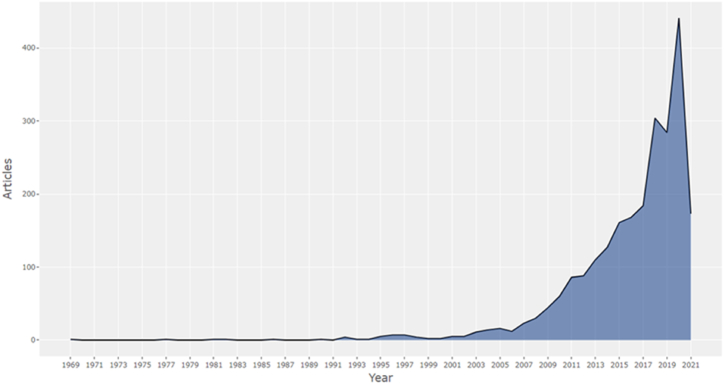
Annual scientific production.

The scientific production collected accumulates with an intensity that grows over time, showing the growing importance of the topics dealt with. According to Fig. 4, at least three distinct stages can be observed.

The initial period leaves a trace of little importance in quantitative terms from the late 1960s (the first item collected is from 1969) until the 1990s of the last century, associated with the emergence of this literature. This initial stage is characterized by a scientific production that does not have a remarkable density and by its lack of clear growth. However, in relation to the issue at hand, it can be said that the agreements reached at the Rio Summit in 1992 and the launch of the Kyoto Protocol in 1997, signed later as a part of the agreements from the Rio Summit, serve to establish the reference of a scientific production with some density, at least from a different perspective. It deals with environmental quality and natural resources management and, more specifically, the more assessed uses of a planetary public good such as the Earth's atmosphere. We could truly assume that in 1992 the specific economic literature on climate change began to grow clearly but not with the intensity of the second phase.

The second phase covers the Great Recession and the first phase of economic recovery up to 2015. In this phase, scientific production related to the terms chosen soared. Two relevant circumstances coincide in this period. First, an international crisis of financial origin is triggered, dominated by a certain inability to manage excessive systemic risk [22]. Second, the effective implementation of the Kyoto Protocol started in 2005 with an experimental phase up to 2007, but with prices and bounded assignments of emission rights in 2008, with organized markets to fix the price of emissions, and mobilization of important volumes of financing, since more restrictive uses of the atmosphere are demanded and, consequently, a change in the structures of production, consumption, and distribution.

The third period, from 2015 to 2021, began with the 2015 Paris Agreement. Since then, many countries have been deploying a more ambitious strategy, often referred to as the energy transition or ecological transition model. It aims to decarbonize the economy (or carbon neutral economy) by 2050, with intermediate targets, mainly for 2030, major changes in production, distribution, and consumption patterns relating to the use of natural resources and the causes, especially, of the so-called climate change. In this third stage, scientific production is accelerating in relation to the second phase, and it does not appear that the hiatus caused by the Covid-19 pandemic has slowed down this evolution.

### 3.3 Applying NLP to the climate change economics literature

**Fig. 5:**
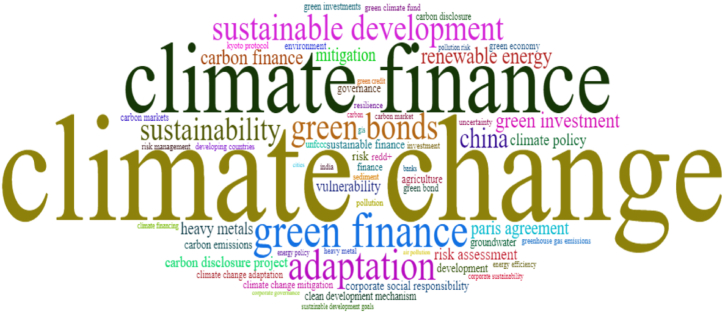
Keyword cloud.

Based on terms drawn from the economic literature and associated with environmental sustainability and climate change, we first proposed a cloud of the 100 most common keywords in the analyzed papers (Fig. 5). The bigram “climate change” is the central term, and “climate finance” is, by far, the second most frequent keyword. Moreover, terms close to “climate finance,” such as “green finance” or “green bonds,” appear strongly in the word cloud. Other general terms of great importance include “adaptation” or “sustainable development” or even “sustainability.” In the third level, words and bigrams such as “renewable energy,” “green investment,” “carbon finance,” “risk assessment,” or “mitigation” address all economic research concerns in this field.

**Fig. 6:**
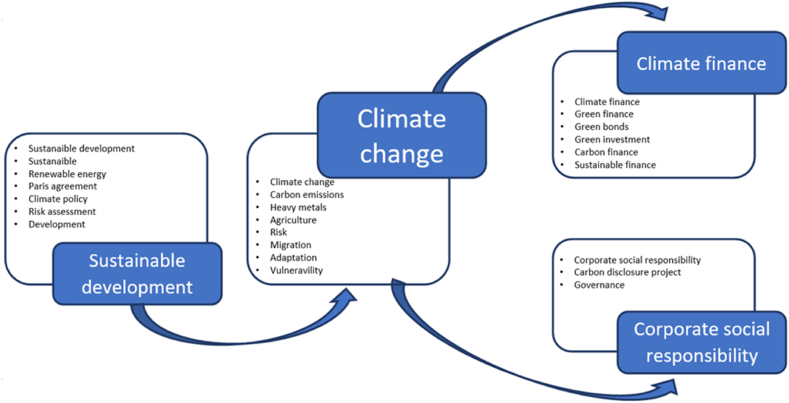
Keyword clusters.

However, to refine the analysis and be more systematic, it may be useful to bring together the keywords according to a relational model (see Fig. 6), where each box collects keywords belonging to the same semantic field. This grouping clarifies that the analysis focuses on the problem to be solved—climate change—to know what produces it (carbon emissions) and the type of problem it generates. It also focuses on the appearance of “risk” and “vulnerability” with a notable effect on some basic activities, such as “agriculture,” and the need for both “mitigation” and “adaptation.” “Heavy metals” are included as a prominent element that allows the synthesis of the environmental issues of unsustainable patterns of production, distribution, and consumption, given that their presence in different natural environments (water, atmosphere, and soil) limits or eliminates their possible uses and consequent loss of basic resources.

However, “climate change” is a specific scientific issue, generally addressed with solutions such as “sustainable development,” which can be disintegrated into the more general terms of “sustainability” and “development.” Furthermore, sustainable development has, because of its frequency, two pivotal elements: “renewable energy” and “climate policy.” These concepts are embodied in the “Paris Agreement,” as the main tools to fight against “climate change,” based on the “risk assessment” of the problem.

This scheme imposes a somewhat reductionist objective in terms of the complex nature of sustainable development, and the possible strategies for achieving it. Nevertheless, this conclusion is given by an initial result of both the search and grouping of the most frequent terms by semantic fields, facilitating ease of analysis.

From this viewpoint, the text analysis of the documents collected in the dataset used in this study provides a dual perspective. On the one hand, we observe that literature is focused onthe private sector. It is necessary for companies to be aware of the change and to see how it is channeled. In this respect, there are a whole of papers where research indicates the long-standing concept of “corporate social responsibility,” which is associated with the response and approach of corporate governance toward the climate crisis.

Given the existence of a periodic measurement instrument that has become increasingly widespread, such as the carbon disclosure project, the research aims to study how these changes are undertaken in the business sector and by whom. This is observed through corporate commitments such as the “carbon disclosure project.”

From a different perspective, there is an important part of the literature analyzed which considers that, meaningful changes in the production and consumption structure for “sustainable development” and the effective fight against “climate change” require strong financing and the development and consolidation of “climate finance.” The problem is the appropriate creation of incentives, products, and markets (“green finance,” “green bonds,” and “carbon finance”) capable of adequately redirecting these financial flows toward the desired objective, while ensuring that all “green investment” provides a combination of returns and risk in line with other investments.

Therefore, based on the frequency of the terms observed in keywords and abstracts, the selected bibliography transcends the initial terms selected by the scientometric researcher. Simultaneously, it enables the classification of a relatively structured and specialized literature. However, we have not provided an explanation of how this literature has evolved and whether the topics covered have changed over time. The literature on climate change began as one of the topics of the 1992 Rio Summit and the resulting international strategy to combat global warming (once 1997 Kyoto Protocol was signed). Therefore, it may be interesting to use dynamic semantic clustering based on the most frequent words found in the abstracts.

### 3.4 Dynamic cluster analysis

The dynamic analysis can be further developed by interpreting the evolution of semantic clusters, as a combination of the density and centrality of the clustering, as displayed in the flow chart above. Following Callon, Courtial, and Laville [15], the joint use of certain terms allows us to determine their position as a combination of centrality to infer the coherence of the cluster and its capacity for development.

**Fig. 7:**
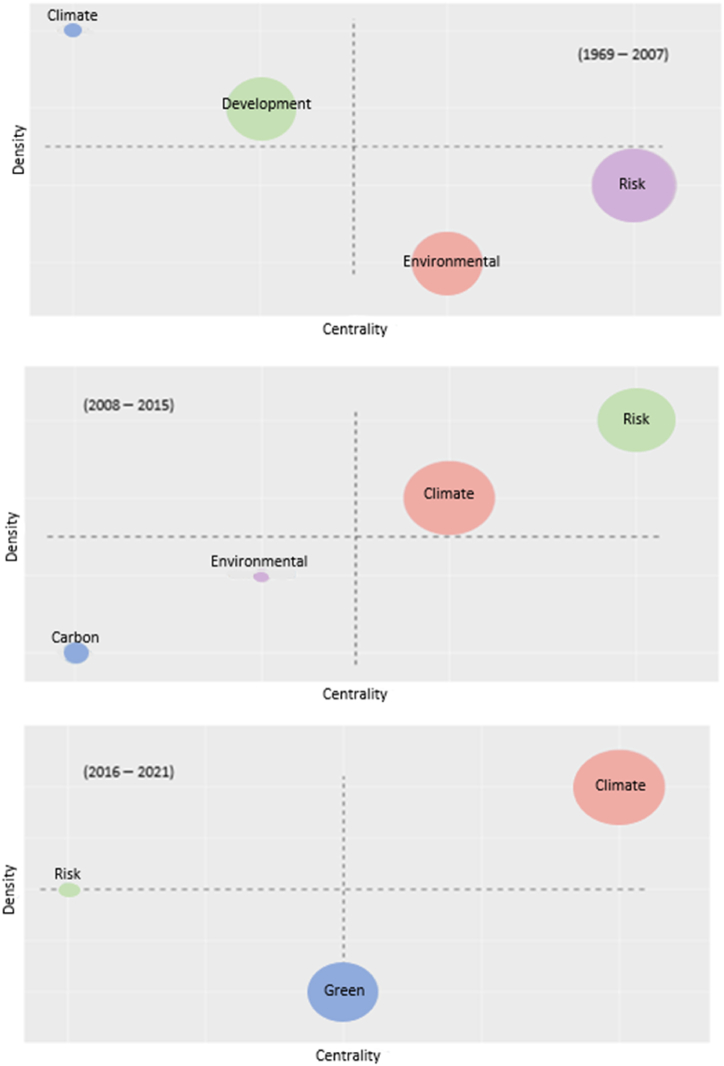
Centrality and density for the timespan.

In the quadrant system proposed by the abovementioned authors (Fig. 7), a substantial part of the economic literature associated with environmental issues focuses on economic development problems, which is an open research agenda with a long tradition in economics. However, it is not yet a central line of research. It coexists with an emerging environmental and “risk” literature, which is more central than the “development” issue.

In the second phase of this evolution (Kyoto Protocol fully enters into force), an important change takes place. Over effects “development” is no longer a subject that condenses the research related to climate change. However, the term “environmental” increases in density and loses much of its centrality. In this phase, the term “carbon” appears as an emerging theme, but with marginal centrality. “Climate” has high centrality in this phase and acquires notable development in the literature, similar to “risk,” which becomes the most developed and central issue.

Finally, in the third phase, from 2015 onward, “climate” is the central and most developed issue. There is a monographic spirit of economic researchers on these issues. “Risk” is a frequent theme in many research studies; however, it has lost all centrality in the previous stage. “Green” is also an emerging but central theme in the latest phase.

### 3.5 Citation analysis

Hitherto, from the keywords and abstracts of the selected documents, remarkable evolution can be observed in the terms that have become increasingly important in this literature stream. “Climate change,” associated with “risk,” forms the bulk of the concerns. “Green” includes complex topics ranging from national development strategies to the financing of “green” projects, including important corporate changes in companies facing the “carbon” problem. This approach is useful, although it is insufficient in advancing the classification and characterization of this economic literature. For this reason, a direct analysis of the contents of this research direction is proposed, starting with a critical reading of the most cited texts, allowing for grouping and classification.

We focus on the contributions with more than 100 citations, as of March 2021. We found only 30 papers that meet this condition (Table 2), slightly more than 1% of the documents in the sample. Based on the central issues in climate change and pollution, the 30 most cited papers can be grouped into four distinct clusters.

**Table 2:** Most cited documents.

Ranked by the Total Number of Citations			Ranked by the Total Number of Citations per year		
Paper	Total Citations	TC per Year	Paper	Total Citations	TC per Year
KOLK A, 2008, EUR ACC REV	356	25.4	LIAO L, 2015, BR ACCOUNT REV	306	43.7
HEINKEL R, 2001, J FINANC QUANT ANAL	315	15	NOBRE CA, 2016, PROC NATL ACAD SCI U S A	184	30.7
LIAO L, 2015, BR ACCOUNT REV	306	43.7	BEN-AMAR W, 2017, J BUS ETHICS	153	30.6
O'BRIEN G, 2006, DISASTERS	253	15.8	STEIGER R, 2019, CURR ISSUES TOUR	77	25.7
DRUCKMAN A, 2011, ENERGY POLICY	221	20.1	MCCOLLUM DL, 2018, NAT ENERGY	102	25.5
STANNY E, 2008, CORP SOC RESPONSIB ENVIRON MANAGE	206	14.7	KOLK A, 2008, EUR ACC REV	356	25.4
REVELL A, 2010, BUS STRATEGY ENVIRON	194	16.2	ZERBIB OD, 2019, J BANK FINANC	71	23.7
REVI A, 2008, ENVIRON URBAN	193	13.8	CAMPIGLIO E, 2016, ECOL ECON	128	21.3
MCKAY PF, 2008, PERS PSYCHOL	187	13.4	DRUCKMAN A, 2011, ENERGY POLICY	221	201
NOBRE CA, 2016, PROC NATL ACAD SCI U S A	184	30.7	LI D, 2018, J BUS ETHICS	79	19.7
LIU G, 2011, ENERGY FUELS	179	16.3	LEWIS BW, 2014, STRATEGIC MANAGE J	157	19.6
MILLER N, 2008, J REAL ESTATE PORTF MANAGE	178	12.7	LIAO X, 2018, ENERGY POLICY	77	19.3
SMITH S, 2010, AUSTRALAS MARK J	177	14.7	ROBIOU DU PONT Y, 2017, NAT CLIM CHANGE	94	18.8
WEINHOFER G, 2010, BUS STRATEGY ENVIRON	158	13.2	TAGHIZADEH-HESARY F, 2019, FINAN RES LETT	52	17.3
LEWIS BW, 2014, STRATEGIC MANAGE J	157	19.6	ONDRACZEK J, 2015, RENEW ENERGY	118	16.8
BEN-AMAR W, 2017, J BUS ETHICS	153	30.6	SAWUT R, 2018, SCI TOTAL ENVIRON	66	16.5
QIU Q, 2011, FOOD CHEM TOXICOL	146	13.3	JUNG J, 2018, J BUS ETHICS	66	16.5
LUO L, 2012, J INT FINANC MANAGE ACCOUNT	143	14.3	LIU G, 2011, ENERGY FUELS	179	16.3
RANKIN M, 2011, ACCOUNT AUDIT ACCOUNT J	141	12.8	REVELL A, 2010, BUS STRATEGY ENVIRON	194	16.2
HAACK P, 2012, ORGAN STUD	129	12.9	O'BRIEN G, 2006, DISASTERS	253	15.8
CAMPIGLIO E, 2016, ECOL ECON	128	21.3	BRUCE N, 2015, ATMOS ENVIRON	109	15.6
HOFFMANN VH, 2007, EUR MANAGE J	127	8.5	DIETZ S, 2016, NAT CLIM CHANGE	93	15.5
ONDRACZEK J, 2015, RENEW ENERGY	118	16.8	TANG DY, 2020, J CORP FINANC	31	15.5
ZIEGLER AD, 2012, GLOBAL CHANGE BIOL	118	11.8	LAPWORTH DJ, 2017, HYDROGEOL J	76	15.2
VANDERMEULEN V, 2011, LANDSC URBAN PLANN	113	10.3	HEINKEL R, 2001, J FINANC QUANT ANAL	315	15
MACREADIE PI, 2014, MAR POLLUT BULL	112	14	SMITH S, 2010, AUSTRALAS MARK J	177	14.7
SÁNCHEZ-AVILA J, 2012, ENVIRON INT	110	11	STANNY E, 2008, CORP SOC RESPONSIB ENVIRON MANAGE	206	14.7
BRUCE N, 2015, ATMOS ENVIRON	109	15.6	LUO L, 2012, J INT FINANC MANAGE ACCOUNT	143	14.3
GUALDI S, 2013, BULL AM METEOROL SOC	108	12	WYLIE L, 2016, MAR POLICY	85	14.2
MCCOLLUM DL, 2018, NAT ENERGY	102	25.5	MACREADIE PI, 2014, MAR POLLUT BULL	112	14

As the first draft of a possible taxonomy of the economic literature, based on the direct reading of the most cited research papers, the grouping mainly revolved around the business literature on how different types of companies tackle the fight against climate change. However, the most cited studies were concerned with specific or general aspects regarding what sustainable development should look like in its fight against climate change. Finally, there is the literature direction on “green finance” that stands out.

However, the method, being based on reading papers with high citation levels, partly penalizes the most recent studies.

### 3.6 Co-citation network

To remedy this deficiency, we proposed a different analysis that constructs a network with the 50 most co-cited authors, with a minimum link of two authors per co-citation, following the Louvain method [23]. We then carried out a centrality analysis of the network, which resulted into a more adjusted cluster than the one deduced by the number of citations. From the four centrality coefficients used (degree, betweenness, closeness, and PageRank), the importance of the works in each cluster was analyzed by the other three centrality coefficients.

**Fig. 8:**
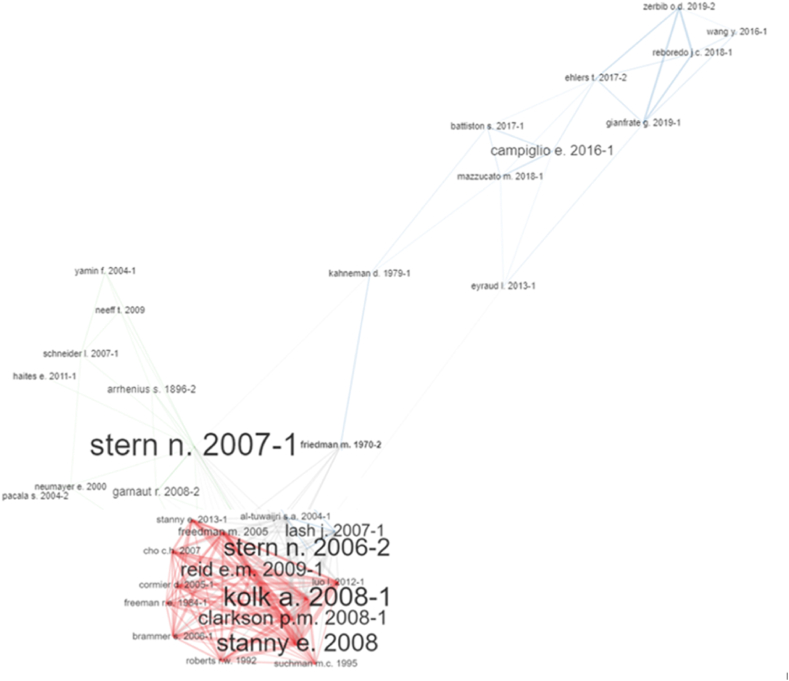
Co-citation network.

The clusters listed below have internal coherence, not only because of the grouping performed by the network analysis but also because of the importance of the resulting coefficients. Therefore, only those clusters with positive betweenness coefficients in more than one case have been highlighted. The minimum betweenness value threshold is set to 100 for the document to be assigned to a cluster. This value is considered good enough to discriminate documents that are distribution points of a community from those that are not. According to Fig. 8, the clusters are as follows:

**Fig. 9:**
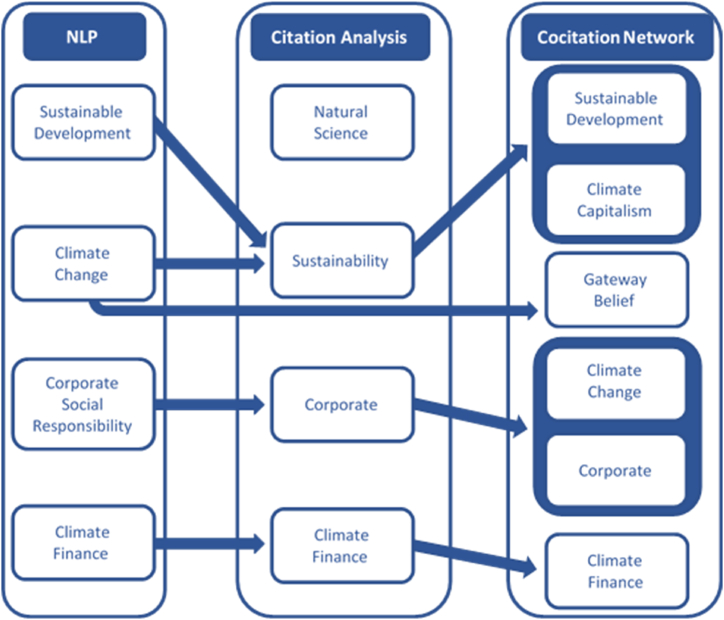
Current state of the literature.

**Cluster 1.** The cluster headed by Kolk [24] responds to the economic literature interested in studying how companies react to climate change, beyond the evolution of public regulation. The co-citation analysis revealed the existence of 47 articles with positive network betweenness and 17 articles with a betweenness equal to or greater than 100. Therefore, 14 of 17 papers have a betweenness degree of greater than 1000. Additionally, five articles published between 2007 and 2011 [[24], [25], [26], [27], [28]] mark the development of this literature, with betweenness values above 500, four of the five having a degree above 2300. In this case, the first two also appear as the most cited in the extracted sample.

**Cluster 2.** Before the formation of Cluster 1, literature around business behavior in relation to pollutant emissions issues was developed. This cluster accumulates 24 papers with a positive betweenness, although only four of them exceed the value of 100. Many papers use Freeman's [29] viewpoint and stakeholder theory, which have been widely used in the development of business ethics research. Others use its counterpoint, Friedman's [30] journalistic article on the pursuit and increase of profit, as the true and sole social responsibility of business.

In any case, existing literature predates the climate change literature and serves as a reference for its subsequent development, especially the works included in Cluster 1. It is relevant to the seminal texts of Hart and Ahuja [31], in terms of competitiveness and costs of pollution abatement by companies. It also resonates with Heinkel, Kraus, & Zechner [32], on the effect that “green investment” can have on corporate behavior, which is a prelude to the sustainable finance literature. Although the latter case is relegated to the cluster due to its poor betweenness value (just over 9), it is the second most cited work in the sample.

**Cluster 3.** This cluster revolves around the financial literature, dominated by regulatory and macroeconomic aspects of sustainable finance and the development and characteristics of green bond markets. In this cluster, 23 papers have positive betweenness values and 10 have values above 100. However, only the first paper exceeds a betweenness value of 500, and there are four others with values above 200.

Kahneman's [33] paper in the *Journal of Econometrics* is very influential here. This is because of its betweenness (higher than 1700) value and appearance, owed to its focus on a different path and unique tools in risk analysis using “prospect theory”. This new theory is critical to the expected utility theory. In prospect theory, there are required characteristics associated with the decision-makers playing an important role because they introduce different psychological perceptions regarding gain and loss under specific circumstances. More importantly, it helps to choose the best financial option among a limited set of possibilities by evaluating multiple biological or psychological attributes of the decision maker. In the context of the climate crisis and the ambitious goals of the Paris Agreement, a new type of literature that connects financial literature that has assumed prospect theory postulates with the climate crisis, has been developed.

Based on these influences, the most recent climate finance literature seems undeveloped and is outside the 30 most cited articles in our sample. It pivots on the seminal article of Ehlers and Packer [34] on “green bonds” by the value of betweenness (higher than 400) or Mazzucato [35], which is dedicated to the macroeconomic effects of financial investments on climate risk. We can also add Reboredo [36] to the green bonds’ literature along with Monasterolo and Raberto [37] and Battiston, Mandel, Monasterolo, Schütze, and Visentin [38], focusing on financial investments associated with climate risk.

**Cluster 4.** The fact that there are four papers with betweenness values above 900 and the Stern and Stern's [39] paper with a high betweenness of almost 5600 points, is explained by their seminal roles in the development of the economics of climate change. The works of Schneider [40] and Bowen, Fankhauser, and Best [41] are highly relevant to the development of specific aspects of climate finance in developing countries.

Alongside these articles, many authors cite Arrhenius [42], who published more than 100 years ago. This study explored the influence of atmospheric carbon on the earth's surface temperatures and quantified them for the first time. A prominent observation is that none of the works in this cluster have more than 100 citations in the sample, although this list includes articles on similar topics, related to sustainable development strategy in certain natural, environmental, or national conditions.

**Cluster 5.** This cluster is characterized by a critique termed “climate capitalism.” We found 13 published research studies with a positive betweenness value, and 8 of them have values above 100. These are systematic works, including analyses and perspectives of contemporary creations such as emissions markets [43], criticisms on the creation of such markets as instruments for the commercialization of carbon “dumps” [44], and alibis— “global warming”—for the transformation and extension of the global economy (climate capitalism) [45]. The final comment on Cluster 4 is also applicable here, given the thematic proximity between Clusters 4 and 5 and the most cited articles referring to sustainable development. Less than 5 articles in this cluster are among those with more than 100 citations; however, some of them should be considered central because of the observed importance of their degrees, combined with their PageRank.

**Cluster 6.** This grouping uses a different perspective. The papers analyze economic behavior based on variables such as vulnerability and adaptability related to the perception of climate change by human communities and their capacity to orientate and support certain policies for the fight against climate change. Only five papers have a betweenness value higher than 100, some of them are very recent, and none have more than 100 citations.

Smit and Wandel [46] have a high degree and are cited almost 6000 times in Google Scholar. Its seminal character is assumed because it is one of the first studies to model the feelings of vulnerability and adaptive capacity of human communities, to global phenomena such as climate change. Similarly, by analyzing almost 2000 individuals in the UK in 2010, Spence et al. [44] show how the public's perceptions of the problem, change radically when there is first-hand experience of climate change. Their analysis method is supported by high betweenness and PageRank values. The “gateway belief” model developed by Van der Linden, Sander, Leiserowitz, Feinberg, and Maibach [47] has also become a reference text. This experiment with more than 1000 individuals shows that when there is an increase in the public's perceptions and scientific consensus on the existence of climate change, the belief that climate change is actively present and threatening is statistically significant. All these results indicate increasing support for public action on the issue.

## 4 Discussion

The aim of this study is to examine how several events, such as the 1992 UN-Rio Summit, the 1997 Kyoto Protocol, and the 2015 Paris Agreement to combat climate change, have fueled rampant and specialized economic literature that has not yet been systematically classified, ordered, and distinguished from the more traditional literature in this field. Using different analysis methodologies does not prevent the establishment of a summary classification, amalgamating the different perspectives in the research, and presenting them coherently. Fig. 8 presents the final outline of the current state of this specialized literature Fig. 9.

Although there is a well-defined economic literature on business and climate change in the 1990s, which can be called the climate change corporate literature or the sustainable corporate literature, it has a more microeconomic, institutional, or business organization orientation. It is combined with new theories such as Freeman's [29] stakeholder theory. Therefore, it strengthens the new perspective of corporate social responsibility management of a business.

A particular confluence is observed between the review based on keyword clustering, several citations, and network theory applied to co-citation. However, in terms of the number of citations, Clarkson et al. [25] would be eliminated because it becomes a seminal article only when the network methodology is used. Nevertheless, this work combined with other most cited studies, for instance, the seminal and influential articles by Kolk [24] and Stanny and Ely [28]0, investigate types of companies concerning their extent of adaptation and affectation to the climate issue, in terms of competitiveness and costs.

Although the clustering points to two different groupings, the contributions in the Cluster 2 network can be subsumed into the sustainable corporate literature. This is because their importance in co-citation is based not on their approach to specific issues in this literature but because of their more seminal and general nature within the corporate literature [[29], [30], [31],^48^]. These studies have served to develop the sustainable corporate literature itself.

Alternatively, the results provide a clearly defined sustainable development literature, fundamentally oriented toward the macroeconomy and aiming to establish the basis for reforming the economic system and making it compatible with climate stability and environmental sustainability. As such, the climate capitalism literature is led by two different approaches. The first one is led by the seminal work of Stern et al. [49] (2006) on the reformist economics of climate change. In the second line of research, several papers [45]a emphasize the limits of the system and its reinvention to adapt, for example, to the development of markets for Green House Gas (GHG) emission rights [43] or in the reformulation of the economic model for survival [50].

In the climate capitalism literature, there is also a convergence in the results obtained by applying different methodologies. Therefore, the use of co-citation networks leads to a dual clustering, in line with the previous paragraph, while the alternative of analyzing the number of citations provides a much more diverse literature, as the most outstanding contributions advocate more specific analyses on how to address the sustainable transition of the system. Furthermore, this orientation appears in the grouping of “climate change” based on keywords.

The third line of research, perfectly defined over time, revolves around sustainable finance literature. This includes a good number of very young contributions concentrated, with some exceptions, between 2012 and 2019. The analysis of networks returned two fundamental research lines: one related to the effects that the destination of the financing of certain investments has in terms of climate risk and the other focusing on the analysis of the green bond markets.

A fourth orientation corresponds to the literature on the public perception of climate change, which we have termed the “gateway belief literature.” It is based on the perception that climate change is associated with human vulnerability and adaptability, and the direct assumption that the problem needs to be assumed as a political priority and resolved in the shortest possible time. This literature stream published its most striking articles between 2006 and 2015.

## 5 Conclusions

In a world where applied research is rapidly expanding and articles are published in the hundreds of thousands, classifying the literature around several concepts is necessary to facilitate focus and specialization in the field of climate change. Therefore, this study can be of great use for researchers interested in this subject and for educators interested in conveying the state of the art. The contribution of this research is the identification and classification of the economic literature arising from international agreements in the fight against climate change, such as the 1992 Rio Summit, the 1997 Kyoto Protocol, and the 2015 Paris Agreement.

For this purpose, the literature has been systematically reviewed, based on bibliometric and scientometric analysis, consisting of techniques such as NLP, conceptual analysis based on clustering techniques, and network theory. This methodology was applied to perform a Boolean search on the Scopus database, which generated a sample of 2444 scientific documents.

Answering the first research question we proposed, it has been possible to review consolidated and differentiated literature on climate change. Overall, the importance of this work lies in clearly identifying four emerging research fields with their own identities: climate capitalism, sustainable corporate, gateway belief, and climate finance.

Answering the second research question, the clustering of the literature has made it possible to identify a specialized field of sustainable development, in which the multisystemic view has been lost. It monopolizes a large part of the research on climate change, to the detriment of other environmental economics topics. Therefore, the importance given to the fight against climate change has promoted a reductionist view of environmental problems.

The third proposed research question is answered, given that this classification yields a differentiated profile for corporate literature on sustainability, which has modified the more traditional conception of corporate social responsibility.

Finally, this study has made it possible to distinguish the very young field of research focused on the development of finance and associated with the fight against climate change. This field, called green finance, clearly answers our fourth research question.

In short, these findings facilitate the development of future research by implementing these identities in established research fields to quickly identify relevant characteristics and limitations.

Likewise, the limitation of this study is that it analyzed documents only from the Scopus database, although it widely covers publications in the scientific field. The details of data [21] and code used [11,12] in this work are provided for replication. As such, despite having characterized and identified the areas of research, it is necessary to continue deepening the systematization to induce effective policy responses in the fight against climate change. This is the result of our partial findings.

## Declaration of competing interest

The authors declare that they have no known competing financial interests or personal relationships that could have appeared to influence the work reported in this paper.
